# Impacts of biogeographic history and marginal population genetics on species range limits: a case study of *Liriodendron chinense*

**DOI:** 10.1038/srep25632

**Published:** 2016-05-10

**Authors:** Aihong Yang, Christopher W. Dick, Xiaohong Yao, Hongwen Huang

**Affiliations:** 1 Key Laboratory of Plant Germplasm Enhancement and Speciality Agriculture, Wuhan Botanical Garden, Chinese Academy of Sciences, Wuhan 430074, Hubei, China; 2 Department of Ecology and Evolutionary Biology, University of Michigan, Ann Arbor, MI 48109-1048, USA

## Abstract

Species ranges are influenced by past climate oscillations, geographical constraints, and adaptive potential to colonize novel habitats at range limits. This study used *Liriodendron chinense,* an important temperate Asian tree species, as a model system to evaluate the roles of biogeographic history and marginal population genetics in determining range limits. We examined the demographic history and genetic diversity of 29 *L. chinense* populations using both chloroplast and nuclear microsatellite loci. Significant phylogeographic structure was recovered with haplotype clusters coinciding with major mountain regions. Long-term demographical stability was suggested by mismatch distribution analyses, neutrality tests, and ecological niche models (ENM) and suggested the existence of LGM refuges within mountain regions. Differences in genetic diversity between central and marginal populations were not significant for either genomic region. However, asymmetrical gene flow was inferred from central populations to marginal populations, which could potentially limit range adaptation and expansion of *L. chinense*.

Species range limits reflect demographic and evolutionary responses to spatially and ecologically varying habitats even in the absence of geographic barriers^1^,^2^,^3^. However, the precise determinants of range limits remain poorly understood^4^,^5^,^6^. From an evolutionary perspective, range limits commonly involve niche constraints^7^ and may be impacted by levels of adaptive genetic diversity and gene flow to marginal populations^3^,^8^. Two relevant hypotheses related to range limits have been proposed.

The central–marginal hypothesis, which is related to the ‘abundant-center’ hypothesis^9^, predicts lower genetic diversity and higher genetic differentiation in marginal populations compared with those in the center of species ranges^10^. Because the highest population density of a species is expected near its geographic center with declining density towards range edges^11^,^12^ (but see Sexton *et al*.^6^), peripheral populations are expected to be smaller, more fragmented, and associated with extremes of suitable habitat^13^. Peripheral populations may suffer from reduced gene flow, strong genetic drift, and low effective population size, leading to lower genetic diversity and higher genetic differentiation than in central populations^10^,^14^. Reduced genetic diversity in turn may limit the adaptive potential for range expansion^15^.

From the perspective of gene flow, the related asymmetrical gene flow hypothesis posits disproportionate levels of gene flow from central to peripheral populations^8^,^16^,^17^. Even though genetic variation from central populations may increase the adaptive genetic diversity of peripheral populations^18^,^19^, central population alleles can swamp locally adaptive variation and inhibit range expansion into novel environments^8^,^16^,^17^. The role of gene flow in shaping range limits is still ambiguous, and empirical tests of the asymmetric gene flow hypothesis are needed^6^,^20^. Hence, estimates of population genetic diversity and gene flow across the central to marginal gradient can provide emprical tests of the processes associated with these two hypotheses and shed light on the evolutionary mechanisms that permit range limit expansions^10^,^21^.

In addition to gene flow, biogeographic history has profoundly influenced species’ geographic distributions and population genetic structure^22^, and adds a layer of complexity to gene-flow based hypotheses. During Quaternary climate oscillations, for example, many temperate species survived in climatically stable refugia^22^. Because of founder effects during post-glacial expansions, genetic diversity has been found to decrease outward from refuge centers along colonization routes^23^,^24^,^25^,^26^. For some species, the highest diversity is found in contact zones where populations from different glacial refugia came into contact^27^,^28^. A combined phylogeographic^29^ and population genetic analysis can help to disentangle historical population dynamics and climatic influences on contemporary genetic and distribution patterns.

Phylogeographic patterns used in identifying refugia and colonization histories have been extensively reported in Europe^30^ and in Northern America^31^, but they have received less attention in East Asia^32^,^33^. East Asian temperate and subtropical forests harbor great species diversity due in part to their proximity to species-rich subtropical and tropical forests, and the regionally complex topography, which provided relative habitat stability through glacial periods^22^,^34^,^35^. The subtropical forests of South-Central China are classified as a global “biodiversity hotspot” because of the large numbers of endemic plant species^36^.

Here, we focus on a Tertiary relict species in this region, *Liriodendron chinense* (Hemsl.) Sarg. (Magnoliaceae), also known as the Chinese tulip tree. The most recent common ancestor of *L. chinense* and its North American sister species, *Lirodendron tulipifera*, had a broad Northern Hemisphere distribution during the globally warmer Mid-Tertiary period, and contracted into its current range disjunction, leading to allopatric divergence of the two *Liriodendron* species in the mid Miocene (*ca.* 14 mya)^37^. *Liriodendron* is comprised of tall deciduous tree species and are shade intolerant. The two species can hybridize under experimental conditions. *Liriodendron tulipifera* is a dominant species in early succession and is widespread in eastern Northern American broad-leaf forests, with its greatest abundance in the southern Appalachian uplands^38^. *Liriodendron chinense*, in contrast, although with similarly wide geographical range, is scattered in subtropical China extending to Northern Vietnam mostly at elevations around 450–1800 m^39^. The scattered distribution of *L. chinense* makes it an ideal case study of range limit dynamics.

The measure of geographic genetic diversity of *L. chinense* had previously been constrained by low numbers of sampled wild populations^40^. In this study, we combined nuclear and chloroplast microsatellite markers and ecological niche modeling (ENM) to evaluate the role of biogeographic history and range margin genetics in determining species range limits of *L. chinense*. Specifically, we aimed to: (i) infer genetic signatures of refugia and possible colonization during the past climate oscillations; (ii) investigate genetic structure and genetic diversity across the whole geographic range; (iii) evaluate the central-marginal and asymmetry gene flow hypotheses. In addition, we sampled haplotypes from the North American tulip tree *L. tulipera* in order to compare the demographic histories within this iconic disjunction between forests of eastern North America and eastern Asia.

## Results

### Chloroplast haplotype diversity and distribution

**Figure 1:**
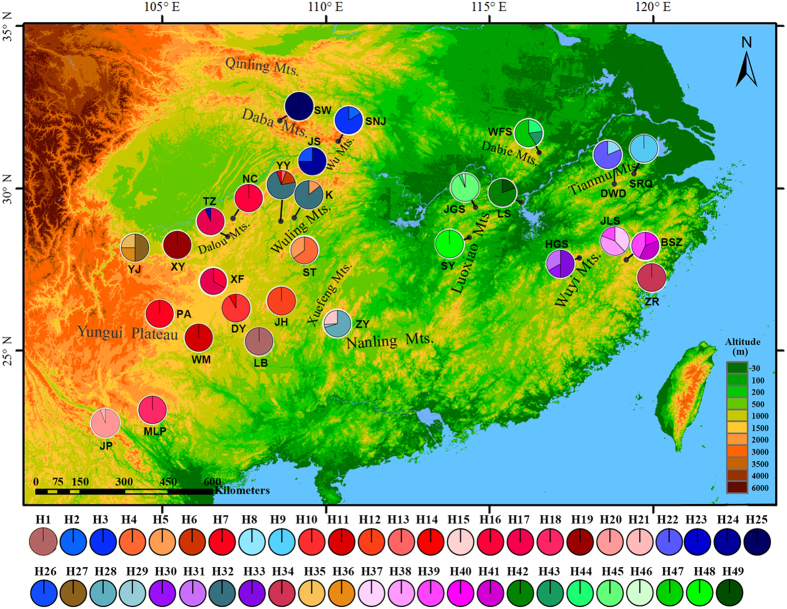
Geographic distribution of cpDNA haplotypes detected in 29 natural populations of *Liriodendron chinense* in China. The map was created by AHY using ArcGIS 9.3 and modified in ArcSoft^®^ PhotoStudio 6.0.

A total of 64 alleles were detected over the 10 polymorphic cpSSR loci in 29 natural populations of *L. chinense* (Fig. 1). The loci exhibited continuous length variation expected from a stepwise mutational model. While Lcp5 contained a 15 bp deletion in the flanking regions, this INDEL did not mask its SSR variant pattern; hence, it was taken as another locus named Lcp5-2. The combination of alleles yielded a total of 49 different unique haplotypes (H1–H49) in *L. chinense* (Table 1) and 13 additional unique haplotypes (H50–H62) in *L. tulipifera* (Supplementary Table S1). Most haplotypes (42 out of 49) in *L. chinense* were population specific, while the remaining seven haplotypes occurred in two adjacent populations (Table 1). The total haplotype diversity in *L. chinense* was high (*h*_T_ = 0.996), whereas the average haplotype diversity within population was relatively low (*h*_S_ = 0.259). The highest population haplotype richness was in population YY located in Wuling Mountains (*H*_R_ = 3.194), followed by population HGS in the west Wuyi Mountains (*H*_R_ = 3.000). Population haplotype diversity (*H*) ranged from 0 to 0.667 (Supplementary Table S2).

### Biogeographic patterns

**Figure 2:**
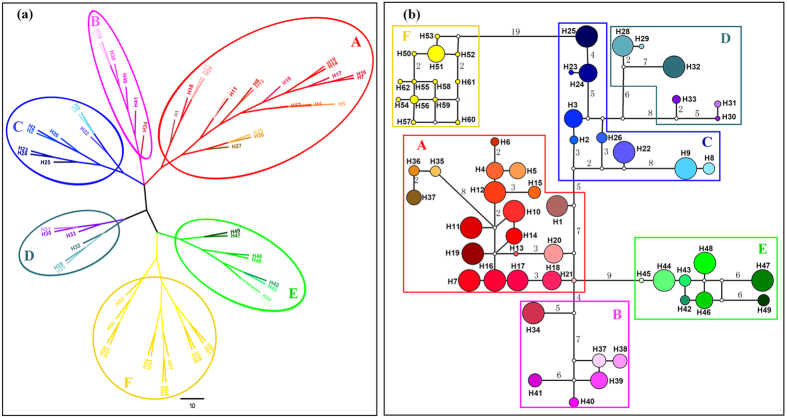
NJ tree (**a**) and Median-Joining network (**b**) for 62 haplotypes of *Liriodendron.* Each numbered circles (H1–H62) represents a unique haplotype, and size of circle corresponding to the frequency of each haplotype; the number besides the line was mutation steps and small open circles indicated the inferred intermediate haplotypes not detected in this investigation. Each haplotype was highlighted with unique color as in Fig. 1 and they were grouped into six clusters (A–F).

*R*_ST_ was significantly higher than *G*_ST_ (*R*_ST_ = 0.901, *G*_ST_ = 0.740; *p* = 0.0004), indicating phylogeographic structure across the range of *L. chinense*. The NJ tree (Fig. 2a) and haplotype network (Fig. 2b) showed a consistent pattern, displaying a distinct geographic distribution that coincided with certain mountain regions (Fig. 1). Closely related haplotypes separated by a few mutational steps were usually limited to adjacent populations within the same mountain regions, while divergent haplotype clusters were found in different regions (Figs 1 and 2).

Cluster *A* contained the largest number of haplotypes and spanned populations in Yungui Plateau and adjacent mountains (Figs 1 and 2). Cluster *E* contained all haplotypes without the INDEL in Lcp5-2 and was restricted to the Dabie and adjacent northern Luoxiao Mountains. The same INDEL deletion in Lcp5-2 was also found in all the sampled individuals of *L. tulipifera*, suggesting the haplotypes of cluster *E* may be ancient. Populations in the northeast region (Tianmu Mountains) harbored haplotypes (H8, H9 and H22) from a separate branch in cluster *C*. Populations in the northwest (Daba Mountains) contained the remaining haplotypes in cluster *C* closest to *L. tulipifera* (cluster *F*). Haplotypes from populations in the east of Wuyi Mountains comprised cluster *B*, while haplotypes from the west of Wuyi Mountains (HGS) formed one branch in cluster *D*. The other branch of cluster *D* was distributed in Wuling (YY, K) and Nanling (ZY) mountain regions, located on the east edge of Yungui Plateau, and also shared haplotypes from cluster *A* with populations in Yungui Plateau.

**Figure 3:**
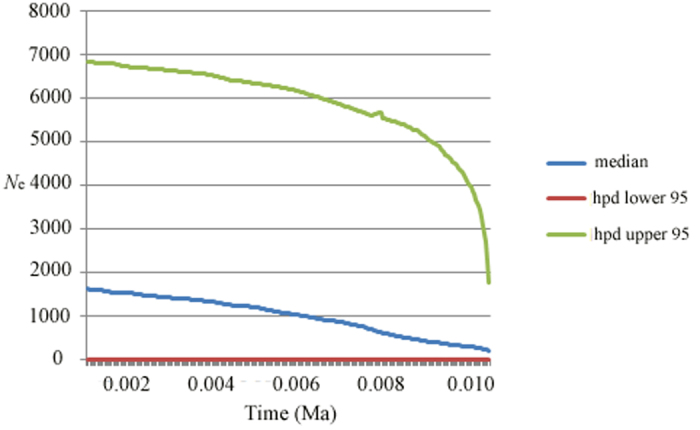
Extended Bayesian Skyline Plots of nuclear microsatellite data from 10 populations of Yungui Plateau.

Though neither SSD and *r* statistics could reject the sudden expansion model (*p* > 0.05), the mismatch distribution pattern over the whole range was multimodal (Table 2), supporting a stable range of *L. chinense*. The absence of significantly negative *F*_S_ also suggested no obvious demographic expansion occurred in this species. Furthermore, separate tests for six refugial regions indicated that *L. chinense* was stable in each refuge except for populations in the Yungui Plateau region, which showed weak signs of demographic expansion (Table 2). Extended Bayesian Skyline Plots showed a signature of a demographic expansion at Yungui Plateau (Fig. 3).

### Nuclear genetic diversity

**Figure 4:**
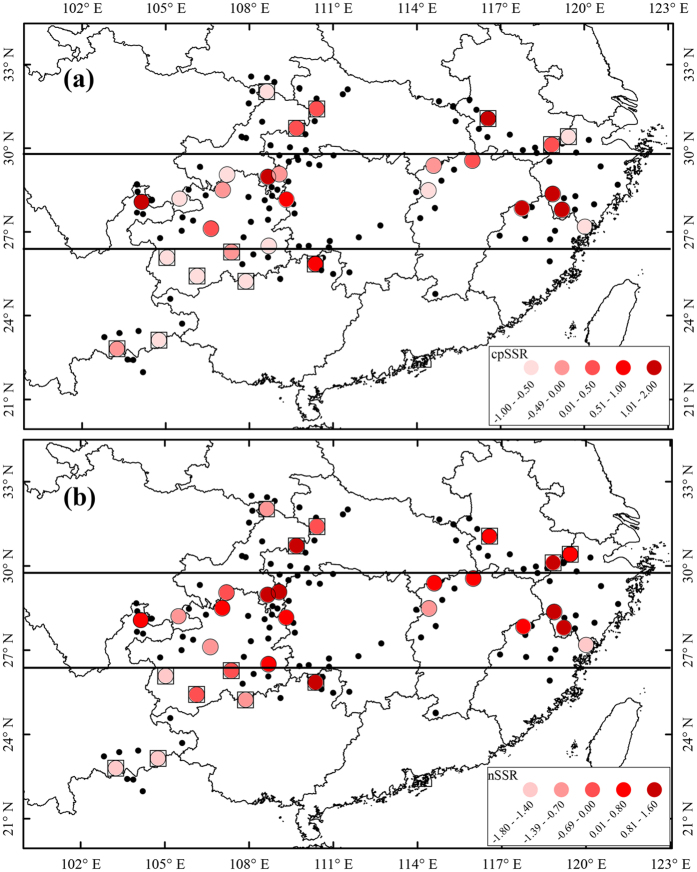
Geographic distribution of haplotype diversity (**a**) and genetic diversity (**b**) for 29 natural populations of *Liriodendron chinense.* Populations with higher genetic diversity were marked in darker red. The central and peripheral populations divided by two horizontal lines were distinguished by round and square edges respectively. The black small dots showed the record occurrence location of *L. chinense*. Maps (**a**,**b**) were generated in ArcMap 9.3 (ESRI Inc).

Eight nuclear microsatellite loci yielded a total of 166 alleles across 693 *L. chinense* individuals. Micro-checker showed no scoring error due to large allele dropout or stutter at any locus. Although 17 out of 812 population-locus pairs deviated from HWE after the sequential Bonferroni correction, only three populations (JS, NC and YJ) exhibited this deviation in two or three loci. Ten population-locus pairs showed significant LD, but no locus pair showed significant LD after Bonferroni correction. Two genetic diversity hotspots were identified: one was along the Wu Mountains and Wuling Mountains toward the western edge of Nanling Mountains; the other was located in the east, near the Tianmu Mountains and northeast of the Wuyi Mountains. Allelic richness (*A*_R_) was highest in population JS (5.185, Supplementary Table S2) located in the Wu Mountains. The highest *H*_E_ was found in population ZY (0.720) in the western Nanling Mountains. The extracted genetic diversity (*H’*_nSSR_) accounted for 90.1% of the five genetic diversity (*N*_A_, *N*_E_, *A*_R_, *H*_O_, *H*_E_) variances. Populations with high genetic diversity typically occurred at mid-latitudes closer to the current northern range edge, and it was obviously low in the southwest ranges (Fig. 4).

### Genetic structure

The standardized genetic differentiation (*G*’_ST_) of *L. chinense* was 0.694 and 0.970 for nSSR and cpSSR markers, respectively, while the genetic differentiation measured as *F*_ST_ was 0.308 and 0.905, respectively. Pairwise *F*_ST_ values were all significant (*p* < 0.05), and ranged from 0.708 to 0.056 for nSSRs, and from 1.000 to 0.258 for cpSSRs. The AMOVA analyses of *L. chinense* revealed most nuclear genetic variation resided within populations (69.17%; Supplementary Table S3), while it only partitioned 9.50% in the chloroplast genome. The genetic variation between east and west regions was considerable in the chloroplast genome (25.67%), but only accounted for a low proportion in the nuclear genome (2.02%).

The STRUCTURE analysis revealed two distinct groups (*K* = 2, Supplementary Fig. S1). Group I included populations WM, MLP, PA, JP, XY and SW, which were exclusively located in the west peripheral ranges. To further explore the substructure of populations in group II, we performed an additional STRUCTURE analysis and recovered two subgroups (IIa and IIb). Populations with assignment proportion lower than 0.8 were referred to as the mixture group (cluster IIm). Populations grouped to cluster IIa were all distributed in the west, and cluster IIb only comprised populations from eastern regions. Cluster IIm comprised five populations (HGS, JS, DY, LB, ZR and TZ) from both regions (Supplementary Fig. S1).

Individual-based assignment analyses using TESS resulted in *K* = 5. Group I of STRUCTURE analysis was split into two subgroups. Group II was divided into three subgroups where one group consisted of individuals from eastern regions, one from western regions and one from both regions (Supplementary Fig. S2). Despite the difference in *K*, the TESS results were largely consistent with STRUCTURE.

### Ecological niche modeling

**Figure 5:**
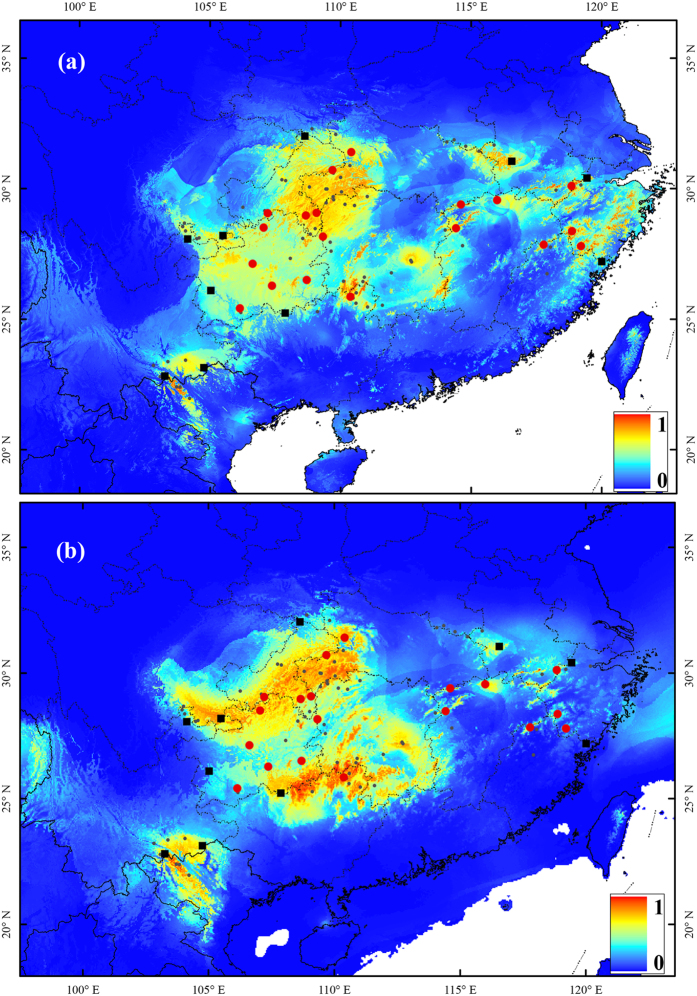
Projected ecological niche models of *Liriodendron chinense* for present (**a**) and LGM (*c*. 21 000 years BP) under MIROC model (**b**). Warmer colors showed areas with better predicted environmental conditions than cooler color. The map was produced by AHY using ArcGIS 9.3.

The predicted models of distribution were significantly better than random (the AUC over ten runs was 0.887 ± 0.044). The predicted present habitat niches nearly fit the actual geographic distribution of *L. chinense* (Fig. 5a). The predicted suitable regions for *L. chinense* during the LGM were almost the same as present and without large scale shifts to the south (Fig. 5b). The continuous west mountain region was suitable for *L. chinense* to survive, especially during the LGM. All current populations are located in or near the predicted suitable regions during LGM, suggesting the possibility of *in situ* refugia of *L. chinense*.

### Genetic comparison between central and peripheral populations

Seven south marginal populations, six north marginal populations and sixteen central populations of *L. chinense* were classified by their geographic locations (Fig. 4; Supplementary Table S2). The environmental suitability of marginal populations was significantly lower than central populations (central: 0.805, marginal: 0.629; *p* = 0.016; Table 3). The population genetic diversity between central and marginal populations was not significant in either genomic region (*p* = 0.090 − 0.882, Table 3; Supplementary Table S2). However, south marginal populations harbored lower genetic diversity than north marginal populations (North: *H*’ =  0.390^a^, South: *H*’ = −0.718^b^, Central: *H*’ =  0.168^ab^, Duncan post hoc test, Table 3). Genetic differentiation (*F*_ST_) in marginal populations was not significantly higher than in central populations in either genome (*p* = 0.199 for nSSR markers and *p* = 0.210 for cpSSR markers, Table 3); it was the highest in the southern marginal populations though still not significant.

### Historical gene flow

Over the whole range of *L. chinense*, the central populations had a significantly higher effective population size than north and south marginal populations, and it was lowest in the south populations (95% CI 4*N*eμ; Central: 0.887–1.060, North: 0.530–0.640, South: 0.427–0.511; Table 4). Asymmetrical gene flow was inferred from central populations to both south and north marginal populations (Table 4); the migration rate from the central to north edges was higher than that from central to the south edge (95% CI: *M*_C→N_ = 16.332–19.651; *M*_N→C_ = 7.726–9.323; *M*_C→S_ = 6.588–8.595, *M*_S→C_ = 3.764–4.901).

## Discussion

### Phylogeographic history of *L. chinense*

The eastern Asian and eastern North American intercontinental floristic disjunction is of longstanding scientific interest^41^. Because of physiographic and climatic differences between the two regions^34^, we expected to encounter distinct phylogeographic patterns in the disjunct *Liriodendron* species despite similarities in ecological niche. In North America, *L. tulipifera*, as a dominant part of the deciduous broadleaf forest, is distributed in higher latitudes and lower altitudes than *L. chinense*, and it experienced a major southward range shift during the Pleistocene glaciations. Genetic and morphological data suggested the existence of isolated refugia in the Apalachicola River basin and in north-central Florida for *L. tulipifera*, while the southeastern coastal plains were implicated as a zone of genetic admixture^38^. In eastern Asia, evidence has accumulated for multiple *in situ* scattered refugia in subtropical China during the LGM^32^,^33^. The ENM-based reconstruction of suitable LGM habitats and demographic tests for *L. chinense* also indicated no sign of large scale habitat shifts to the south and no obvious colonization to the north after the LGM, but instead supported a model of geographic stability, partially leading to lower latitude distribution compared with its sister species in North America.

Geographical haplotype distributions of *L. chinense* suggested at least six scattered subtropical refugia in the Yungui Plateau and the mountains of Daba, Dabie and Northern Luoxiao, Nanling, Tianmu and Wuyi. These putative refugia have been recognized as centers of plant diversity and possible glacial refugia for other ancient relict tree species such as *Taxus wallichiana*^42^, *Ginkgo biloba*^43^ and *Tapiscia sinensis*^44^.

Potential habitat of *L. chinense* in the eastern region remained fragmented and without admixture during the cold period as predicted by ENM and genetic data. Populations in the Dabie and adjacent Luoxiao Mountains harbored a unique haplotype branch with the most mutation steps to other branches, suggesting isolation from other regions. Central populations (JGS) within this region harbored the most diverse and potentially ancestral haplotypes that may have preceded colonization of adjacent regions. The Tianmu and Nanling Mountains have acted as refugia for many temperate tree species including *Ginkgo biloba*^43^ and *Fagus engleriana*^45^. It was also the case in *L. chinense*. The habitat of the western region was continuously ecologically suitable for *L. chinense*^39^, even during the LGM as inferred from the results of ENM. Daba Mountains is considered a center of genetic diversity center and refuge for relict species (e.g. *Tapiscia sinensis*, Zhang *et al*.^44^), where the most putatively ancient haplotypes in *L. chinense* were found. The Yungui Plateau, which extends to highlands in warm Northern Vietnam, was also an important LGM refuge for *L. chinense*. Populations there comprised a cluster without much haplotype loss (cluster *A*), also suggesting that Yungui Plateau was environmentally stable. Populations in the south edge contained putative ancient haplotypes; their expansion to north Yungui Plateau and adjacent mountains was supported by demographic tests, and contributed to the genetic diversity hotspots in the Wuling and Nanling Mountains (Fig. 1), where potential refugia are supported by the ENM. The western Wuyi/Nanling ancestor diverged into two lineages (cluster *D,* Figs 1 and 2); descendants from one branch may have migrated eastward to western Wuyi Mountains (HGS), which shared a closer genetic relationship with western populations in both genomes (Fig. 1; Supplementary Fig. S1), and another branch of descendants likely recolonized along west edges of Nanling Mountains and further migrated northwards, leaving some relicts in Wuling Mountains (Fig. 1). Although this demographic trend was not detected by the mismatch distribution tests, populations along this potential route may have gone extinct due to the invasion of evergreen or coniferous forest during the Quaternary^32^,^35^, and the Nanling Mountains were not climatically suitable for *L. chinense* as projected by the ENM.

### The central-marginal hypothesis

Reduced genetic diversity towards the periphery of the species ranges has been found in many species^13^. For instance, 64.2% of 134 studies detected the expected decline in genetic diversity towards range margins (in review of Eckert *et al*.^9^). However, the distribution of genetic diversity in *L. chinense* did not support the central-marginal hypothesis. Many other factors, such as range geometry and orientation, latitudinal and ecological environmental gradients, and phylogeographic history, may confound central–marginal patterns^9^,^10^,^46^, and is has been shown that the post-glacial range dynamics are the clearest determinants of genetic diversity for many species^23^,^24^,^25^,^26^. Species under recent or ongoing range changes usually deviate from C-M expectations in some core-to-edge transects^47^, and support for C-M processes tend to derive from species with relatively stable geographic ranges^48^. Thus, the central–marginal pattern should be interpreted in the context of phylogeographic history and historical range dynamics^9^. *Liriodendron chinense* survived from the glacial period by splitting into many scattered refugia over the whole range, and it was relatively long-term demographically stable. Hence, as revealed by chloroplast genomic markers, which was less variable and maternally transmitted, marginal populations of *L. chinense* mantained genetic diversity from *in situ* refugia, and had abundant genetic diversity as central populations (Fig. 4; Table 3). Thus, the phylogeographic history of *L. chinense* may largely responsible for its deviation from C-M expectations.

However, populations of *L. chinense* in south edge tended to have higher genetic differentiation and reduced genetic diversity in terms of both allelic richness (*N*_E_) and genetic heterozygosity (*H*_O_, *H*_E_) than north marginal populations. It has been shown that the south margin of temperate species, such as *L. chinense*, functioned as refugia during glacial intervals leading to population differentiation^24^. The northern margin, on the other hand, was more climatically suitable for *L. chinense* and maintenance of within population diversity was favored over population differentiation. Although neutral genetic diversity may have no direct connection with adaptive traits^20^,^49^, the lower diversity at the south margins may be associated with reduced evolutionary potential, which may inhibit *L. chinense* to adapt to novel environments beyond these range limits^1^,^2^,^9^. This may partly explain geographic range limits of *L. chinense* in the south edge of its range.

### The asymmetrical gene flow hypothesis

There are few empirical tests of the assumptions that asymmetrical gene flow may swamp local adaptation in peripheral populations with potentially maladapted genes, thereby precluding range expansion^6^. To explore the role of gene flow in determining range limits, in the present study, we focused on the migration rate in central and marginal populations, a measure describing the effects of immigration on genetic variation in local populations. In the case of *L. chinense*, the asymmetrical gene flow hypothesis was supported and asymmetrical gene flow from central to both south and north marginal edges over the whole range was inferred. Hence, the large influx of foreign individuals may have swamped local adaptive alleles of the edge populations, and impact the range limit of *L. chinense.* However, special attention should be paid to the genetic effects of gene flow to marginal populations apart from the symmetry pattern. The relative contribution of swamping local adaptive alleles and providing potential adaptive alleles for marginal populations was crucial in determining the asymmetry gene flow hypothesis for range limits^8^,^18^. Hence, future studies may consider performing reciprocal crosses between central and marginal populations and compare the performance (population fitness components) of progeny in order to make the connection between gene flow and adaptive limits to range expansion of this species.

In summary, in the present study, we investigated the phylogeographic pattern and genetic structure of *L. chinense* over its geographical range, attempting to uncover the potential impacts of historical climate oscillation on its current spatial genetic structure and evaluate expectations of two longstanding evolutionary hypotheses involving range limits by molecular population genetic investigation. Inferred from the phylogeographic history of *L. chinense*, Quaternary glacial cycles have played a prominent role in shaping the geographic distribution and genetic structure of *L. chinense*. In accordance with most tree species in subtropical forests of China, no massive range shifts but multiple local refugia were suggested^32^ and no obvious range expansion occurred in *L. chinense* after the LGM, resulting a relatively even genetic diversity distribution pattern over the central and marginal populations. The expected asymmetrical gene flow from central to marginal populations was observed in *L. chinense*, which may partly explain the range limits of *L. chinense*. However, whether lower genetic diversity and asymmetrical gene flow is associated with lower population fitness performance beyond the range limits remains uncertain. As a complement to molecular studies, field studies investigating population fitness performance should be used in future studies to provide deeper insight into evolutionary mechanism involved in the maintenance of species’ range limits.

## Methods

### Population sampling

We collected young leaves of 693 individuals from 29 wild populations across the entire range of *L. chinense* in 2013 (Table 1). Fifteen to 35 individuals (mean *N* = 24) for each population were sampled, except for population XF and HGS, from which only nine and six wild individuals were obtained respectively. Twenty-seven *L. tulipifera* samples collected from a mixed population introduced from the U.S. were used for comparative purposes. The genomic DNA was extracted from young leaves using the CTAB method.

### Microsatellite genotyping

Ten mononucleotide chloroplast SSR primers developed for *L. chinense*^50^ (Lcp5, Lcp15, Lcp19, Lcp21, Lcp24, Lcp26, Lcp33, Lcp39, Lcp48 and Lcp49) were used to screen the chloroplast genome. Nuclear genomic variation was analyzed by four *de novo* characterized nuclear microsatellite primers in *L. chinense*^51^ (LC027, LC097, LC120, LC269) and four EST-SSR primers characterized from *L. tulipifera*^52^,^53^ (LT013, LT015, LT026, LT058). PCR amplification was performed according to the published protocols. Alleles were identified by 6% polyacrylamide gel electrophoresis with enhanced silver stain using a 25-bp DNA ladder marker, and meanwhile at least two individuals from each population were selected to be run together to correct all the individual allele scores. To check allele-size variation at the ten chloroplast microsatellite loci, at least one sample of PCR product for each allele at each locus were subjected to DNA sequencing using the ABI 3730xl DNA Analyzer.

### Statistical analysis of chloroplast microsatellite markers

Unique combinations of cpSSR alleles within a chloroplast genome were defined as haplotypes. The number of haplotypes (*N*_A_), effective number of haplotypes (*N*_E_), the haplotype richness (*H*_R_), and the haplotype diversity^54^ (*H*) were calculated using the program GenAlEx 6.5^55^. Since these genetic diversity parameters were highly correlated, we performed a principal component analysis (PCA) in SPSS 13.0 to extract the genetic diversity indices as a single vector (following Garner *et al*.^23^; PC1 eigenvalue = 3.79, variance explained = 94.7%, Supplementary Table S4) and later map these estimates.

The partitioning of genetic variation was examined by hierarchical analysis of molecular variance (AMOVA) under 10 000 permutations in Arlequin version 3.1^56^. The standardized measure of genetic differentiation *G*’_ST_^57^ was calculated in MSA 4.05^58^.

The presence of phylogeographic structure was determined by whether the value of *R*_ST_ (considering the mutational distances between haplotypes) was significantly higher than the *G*_ST_ (depending on frequencies of haplotypes) under 1000 random permutations in PermutCpSSR version 2.0^59^.

A haplotype neighbor joining (NJ) tree was constructed based on 1 000 bootstraps of Nei’s distance^60^ with *L. tulipifera* included as the outgroup using PowerMarker v3.25^61^. Intraspecific genealogies of haplotypes were constructed using a median-joining network in Network 4.610^62^ with all length polymorphism in microsatellite sites included and weighted equally. The demographic history of *L. chinense* was assessed by neutrality tests with Tajima’s *D* and Fu’s *F*s and mismatch distribution analysis (Supplementary Method S1). Chloroplast microsatellite data were coded as “DNA” type according to Pereira *et al*.^63^ by their nucleotide repeat numbers. Tests were examined in two levels: the whole range and each of the six putative refugia regions.

### Statistical analysis for nuclear microsatellite markers

Genotyping errors were identified by the software Micro-checker (available at http://www.norwichresearchpark.com/ourresearch/researchgroups/elsa/software/microchecker.aspx). Linkage disequilibrium (LD) and Hardy-Weinberg equilibrium (HWE) for each population and microsatellite locus pair was assessed in Genepop version 1.2^64^. Population genetic diversity parameter such as effective number of alleles (*N*_E_), allelic richness (*A*_R_), expected genetic diversity (*H*_E_) and observed genetic diversity (*H*_O_) were calculated using the software GeneAlEx 6.5^55^ and FSTAT version 2.9.3^65^.

The extraction of genetic diversity and estimation of population differentiation were performed as described in cpSSR markers (PC1 eigenvalue = 4.51, variance explained = 90.1%, Supplementary Table S5). The population genetic structure of *L. chinense* was determined using STRUCTURE v2.3.3^66^. An admixture ancestry model with correlated allele frequencies and no prior information of population origin was used. Ten independent runs for each *K* (1–33) were carried out by 50 000 burn-in and 100 000 iterations of the MCMC. The suitable number of clusters (*K*) was chosen at the largest rate of change in the log probability of data between successive *K* values^67^.

An additional cluster analysis was performed with TESS v2.0^68^, which detects spatial correlation between individuals. In contrast to STRUCTURE, it assumes that all of the individuals are equally unrelated^68^. The program was run with 100 replicates under the admixture (BYM) model, using 50,000 MCMC sweeps after a burn-in of 10,000 for *K* = 2 to 29 and with a spatial interaction parameter of 0.6. The optimal *K* for the data is at the point where the deviance information criterion (DIC) curve switches from a sharp decrease to a plateau^68^. The 20 runs with lowest DIC scores were combined using software CLUMPP^69^ and the graphical representation was visualized using DISTRUCT 1.1^70^.

Extended Bayesian Skyline Plots (EBSPs) were used to test the hypothesis of demographic expansion at Yungui Plateau. The EBSPs were constructed in BEAST v1.80^71^, and settings were referred to Teske *et al*.^72^. The mutation rate of microsatellites was set as 5 × 10^−4^ per locus per generation^73^. Two independent runs were carried out for a chain length of 1 × 10^8^ and a logging frequency of 1 × 10^6^.

### Ecological niche modeling (ENM)

The ENM of *L. chinense* was carried out to evaluate the habitat suitability at present and during the Last Glacial Maximum (LGM; ~21 000 years BP). One hundred and forty-eight occurrence localities including three from Northern Vietnam (Fig. 4) were obtained by our investigation or from credible herbarium records downloaded from two specimen databases (available at http http://www.cvh.org.cn/; http://www.gbif.org/) and local flora. Nineteen bioclim variables were extracted from the WorldClim database^74^ at 30 arc-seconds resolution for present and 2.5 arc-minutes resolution for LGM climate based on the Model for Interdisciplinary Research on Climate (MIROC) suitable for East Asia. To avoid potential over fitting, seven bioclim variables (bio1, bio2, bio7, bio8, bio12, bio17, bio18) with lower level of correlation (*r*^2^ < 0.75) were used in the climatic niche model building. The ENM was executed using Maxent version 3.3.3k^75^. We randomly partitioned 30% of occurrence records as the test set and used the remaining as training set. Maxent was run using the default settings with ten repeats, and the final maps of prediction results were visualized in arcgis 9.3 (ESRI, Redlands, CA, USA). The projected present habitat suitability scores for each sampled population were extracted.

### Genetic diversity comparison between central and marginal populations

We defined the central and marginal populations of *L. chinense* by their geographic locations. Geographical locations of *L. chinense* based on extensive field surveys and herbarium records were digitized into the range maps in arcgis 10.2. We then divided species’ range into peripheral and central regions by equal area^76^. Two bands were constructed by collapsing the range boundaries with latitude lines from both the south and north range limit until the area occupied achieved the ratio of 1:2:1 (Fig. 4). That is, the population near northern or southern range limits was referred as peripheral population, and populations situated in nearly central parts of the distribution range were defined as central populations. The projected environment suitability and population genetic diversity of central *vs.* marginal populations in both genomes were compared using *t*-tests or Mann-Whitney *U* tests depended on the distributed mode of each parameter (the normal distribution pattern was tested by One-sample Kolmogorov-Smirnov test), and ANOVA was performed to evaluate the difference of central, south marginal and north marginal followed by Duncan post hoc test in SPSS 13.0 program. The genetic differentiation between the two groups was compared using a 1 000 permutation test in the FSTAT version 2.9.3^65^.

### Estimated historical gene flow

Mutation-scaled effective population size (theta = 4*Ne*μ) and directional mutation-scaled effective migration rate (*M* = *m*/μ) were calculated in Migrate v3.5.1 (available at http://popgen.dcd.fsu.edu/Migrate-n.html) using nuclear microsatellite data under the suggested Brownian microsatellite mutation model. Considering different situations along species’ edges^6^ and to achieve the gene flow between central and marginal populations clearly, we grouped all the populations into three meta-populations, i.e. north marginal populations, south marginal populations and central populations with randomly generated a subset of 30 individuals for justification. Most run parameters were left at default values, initial estimates of theta and *M* were generated from *F*_ST_ values, and we conducted searches using ten short chains of 200 000 sampled trees and three long chains each with 70 000 record trees out of 3.5 million sampled trees, and discarded the initial 20 000 trees as burn in.

## Additional Information

**How to cite this article**: Yang, A. *et al*. Impacts of biogeographic history and marginal population genetics on species range limits: a case study of *Liriodendron chinense. Sci. Rep.* **6**, 25632; doi: 10.1038/srep25632 (2016).

## Supplementary Material

Supplementary Information

## Figures and Tables

**Table 1:** Geographical locations of 29 sampled natural populations of *Liriodendron chinense* and population genetic diversity revealed by nSSR and cpSSR markers.

Code	Population location	Mountain region	Altitude	Latitude (° N)	Longitude (° E)	*N*s	*H*’_nSSR_	*H*’_cpSSR_	Haplotype
Western region									
JP	Jinping, Yunnan	Yungui Plateau	1595	22.812	103.257	17	−1.461	−0.393	H20, H21
MLP	Malipo, Yunnan	Yungui Plateau	1683	23.137	104.754	16	−1.767	−0.983	H18
WM	Wangmo, Guizhou	Yungui Plateau	1295	25.407	106.133	32	−0.134	−0.983	H11
LB	Libo, Guizhou	Yungui Plateau	849	25.226	107.868	20	−0.715	−0.983	H1
DY	Duyun, Guizhou	Yungui Plateau	1368	26.270	107.364	24	−0.319	−0.292	H10, H14
JH	Jianhe, Guizhou	Yungui Plateau	1000−1300	26.497	108.690	31	0.171	−0.983	H12
XF	Xifeng, Guizhou	Yungui Plateau	1470	27.119	106.623	9	−0.922	0.411	H16, H17
PA	Pu’an, Guizhou	Yungui Plateau	1614	26.095	105.023	36	−1.627	−0.983	H7
YJ	Yanjin, Yunnan	Yungui Plateau	783	28.067	104.135	20	0.077	1.614	H27, H35, H36
XY	Xuyong, Sichuan	Yungui Plateau	1278	28.197	105.492	33	−0.829	−0.983	H19
TZ	Tongzi, Guizhou	Dalou Mts.	1579	28.500	107.038	15	0.371	−0.352	H17, H23
NC	Nanchuan, Chongqing	Dalou Mts.	1241	29.049	107.198	24	−0.039	−0.980	H16
SW	Chengkou, Chongqing	Daba Mts.	1404	32.030	108.628	33	−0.965	−0.983	H25
SNJ	Shennongjia, Hubei	Daba Mts.	1400	31.401	110.405	18	−0.428	0.042	H2, H3
JS	Jianshi, Hubei	Wu Mts.	1787	30.713	109.680	20	1.446	0.289	H24, H26
YY	Youyang, Chongqing	Wuling Mts.	1329	28.968	108.656	27	1.254	1.846	H6, H13, H14, H32
K	Longshan, Hunan	Wuling Mts.	1200	29.067	109.067	35	1.128	−0.064	H5, H32
ST	Songtao, Guizhou	Wuling Mts.	882	28.157	109.319	20	0.264	0.505	H4, H5
ZY	Ziyuan, Guangxi	Nanling Mts.	1181	25.850	110.363	27	0.996	0.785	H15, H28, H29
Eastern region									
HGS	Yanshan, Jiangxi	Wuyi Mts.	1200–1800	27.841	117.774	6	0.617	1.618	H30, H31, H33
JLS	Suichang, Zhejiang	Wuyi Mts.	600–952	28.361	118.858	21	1.570	1.648	H37, H38, H39
BSZ	Qingyuan, Zhejiang	Wuyi Mts.	1480	27.787	119.198	21	1.038	1.648	H39, H40, H41
ZR	Zherong, Fujian	Wuyi Mts.	438	27.197	119.997	24	−1.690	−0.983	H34
SRQ	Anji, Zhejiang	Tianmu Mts.	931	30.412	119.433	31	0.635	−0.983	H9
DWD	Jixi, Anhui	Tianmu Mts.	1180	30.110	118.835	32	1.538	0.092	H8, H22
WFS	Shucheng, Anhui	Dabie Mts.	810	31.059	116.548	22	0.114	1.340	H42, H43, H46
JGS	Tongshan, Hubei	Luoxiao Mts.	900–1100	29.384	114.602	27	0.018	−0.035	H43, H44, H45
LS	Lushan, Jiangxi	Luoxiao Mts.	1000–1200	29.548	115.987	31	0.546	0.111	H47, H49
SY	Tonggu, Jiangxi	Luoxiao Mts.	230	28.475	114.414	21	−0.886	−0.983	H48

Number of samples in the analysis abbreviated as *Ns, H’*_nSSR_ and *H’*_cpSSR_ represented extracted genetic diversity and haplotype diversity index by principal component analysis, respectively.

**Table 2:** Demographic tests of *Liriodendron chinense.*

Group	Mismatch test		Neutrality tests	
	Modal	SSD (*p*-value)	*r* (*p*-value)	*F*_S_ (*p*-value)
Yugui Plateau	Unimodal	0.012 (0.140)	0.021 (0.179)	−0.146 (0.549)
Daba Mountains	Multimodal	0.085 (0.075)	0.203 (0.015)	6.593 (0.975)
Nanling Mountains	Bimodal	0.424 (0.000)	0.446 (0.947)	7.167 (0.965)
Tianmu Mountains	Bimodal	0.531 (0.000)	0.397 (0.952)	11.965 (0.998)
Dabie-Mufu Mountains	Multimodal	0.077 (0.069)	0.133 (0.015)	4.196 (0.937)
Wuyi Mountains	Multimodal	0.067 (0.029)	0.100 (0.008)	10.516 (0.998)
Whole Range	Multimodal	0.003 (0.706)	0.003 (0.547)	3.252 (0.888)

SSD: the Sum of Square Deviation; *r*: Raggedness index.

**Table 3:** Comparisons of genetic diversity and genetic differentiation between central and marginal populations of *Liriodendron chinense.*

Groups		*N*_S_	Climate suitability	nSSR		cpSSR	
				*H’*	*F*_ST_	*H’*	*F*_ST_
Central		365	0.705^**ab**^	0.168^**ab**^	0.245	0.252	0.720
Marginal	North	156	0.804^**a**^	0.390^**a**^	0.239	−0.034	0.767
	South	172	0.575^**b**^	−0.718^**b**^	0.424	−0.547	0.895
*p*_(C–M)_			0.016	0.151	0.199	0.134	0.210

*N*_S_: Number of sampled individuals; Climate suitability: The present climate suitability for each population predicted by the ecological niche modeling; *H*’: extracted genetic diversity and haplotype diversity index by principal component analysis; *F*_ST_: genetic differentiation.

**Table 4:** Estimates of mutation-scaled effective population size (Theta = 4*N*eμ) and mutation-scaled migration rate (*M*) in each population group in *Liriodendron chinense*.

Group	Theta	Migration rate (*M*)		
		North	Central	South
North	0.467 (0.427–0.511)	–	8.500 (7.726–9.323)	3.380 (2.755–4.093)
Central	0.581 (0.530–0.640)	17.941 (16.332–19.651)	–	7.547 (6.588–8.595)
South	0.968 (0.887–1.060)	5.091 (4.252–6.028)	4.307 (3.764–4.901)	–

Values in brackets represented the 95% confidence values, migration was from the row population to the column population.
