# Supplementary material for: Impacts of biogeographic history and marginal population genetics on species range limits: a case study of Liriodendron chinense
Source: Sci Rep. 2016 May 10;6:25632. doi: 10.1038/srep25632 (PMC4861920; doi:10.1038/srep25632)
Supplement: Supplementary Information [file srep25632-s1.pdf]

Supplementary information for:

Impacts of biogeographic history and marginal population genetics on species range limits: a case study of *Liriodendron chinense*

Aihong Yang[1], Christopher W. Dick[2], Xiaohong Yao[1,*] and Hongwen Huang[1]

[1] *Key Laboratory of Plant Germplasm Enhancement and Speciality Agriculture, Wuhan Botanical Garden, Chinese Academy of Sciences, Wuhan 430074, Hubei, China;*

[2] *Department of Ecology and Evolutionary Biology, University of Michigan, Ann Arbor, MI 48109-1048, USA;*

[*] Corresponding author: Xiaohong Yao, yaox@wbgcas.cn, Tel: +86-27-87510567, fax: +86-27-87510251.

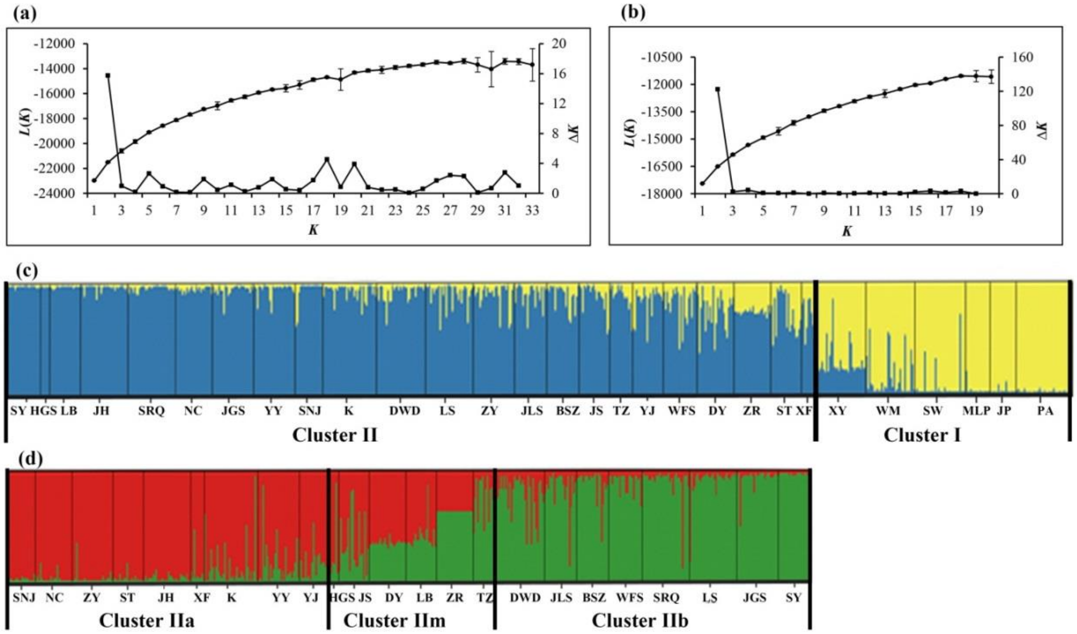

Supplementary Figure S1 Bayesian inference for the determination of suitable cluster (*K*) and the estimated cluster proportion using STRUCTURE for *Liriodendron chinense*. The log probability *L*(*K*) at each *K* (10 replicates) and Δ*K* for all the 29 sampled populations (a) and populations in Cluster II (b) to estimate the suitable cluster. The assignment proportion in all the sampled 29 populations (c) and 23 populations in Cluster II (d), each individual was represented by a thin vertical line, which was partitioned into *K* segments that represent its estimated population group membership fractions.

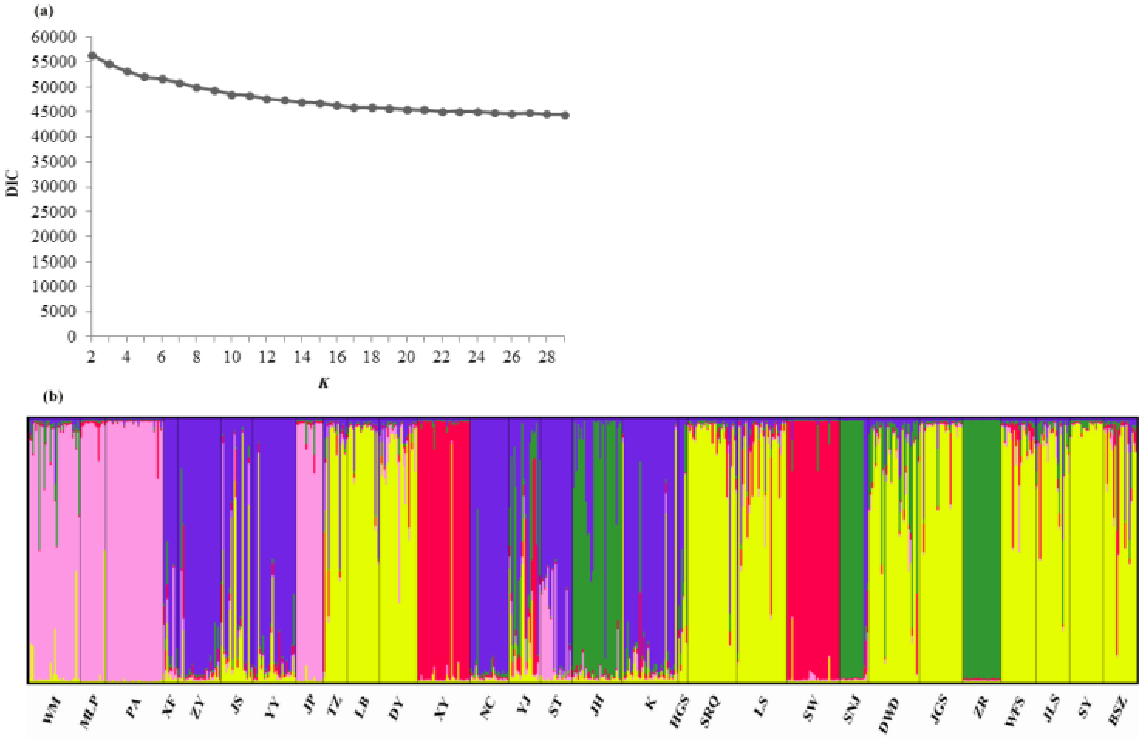

Supplementary Figure S2 Genetic structure of 29 natural populations of *Liriodendron chinense* were estimated using TESS. Mean values of the DIC statistics estimated for models with the number of genetic clusters (*K*) ranging from 2 to 29 suggesting the presence of five clusters (a); Bar plot resulting from TESS when *K* = 5 (b).

Supplementary Table S1 Frequency (No. indiv.) and repeat number of each haplotype in 29 natural populations of *Liriodendron chinense* and one cultivated population of *Liriodendron tulipiphera*. Note: "+" and "–" represent the 15 bp INDEL (TAGATGTAAAATAGA) in locus Lcp5-2 respectively.

Haplotype	No. indiv.	Species	Repeat numbers of each locus										
			Lcp5	Lcp15	Lcp19	Lcp21	Lcp24	Lcp26	Lcp33	Lcp39	Lcp48	Lcp49	Lcp5-2
H1	20	*L. chinense*	17	17	8	11	9	13	14	9	25	12	+
H2	3	*L. chinense*	17	15	8	11	11	13	12	9	27	13	+
H3	15	*L. chinense*	17	15	8	12	11	13	12	9	27	13	+
H4	13	*L. chinense*	15	18	8	12	10	13	15	10	19	10	+
H5	12	*L. chinense*	15	18	8	12	10	13	16	10	19	10	+
H6	3	*L. chinense*	16	17	8	12	10	13	15	10	19	10	+
H7	36	*L. chinense*	17	18	8	12	10	13	13	10	19	10	+
H8	6	*L. chinense*	14	15	8	11	10	14	12	10	23	10	+
H9	31	*L. chinense*	14	15	8	11	10	14	12	10	24	10	+
H10	22	*L. chinense*	15	18	8	12	10	14	13	10	19	11	+
H11	32	*L. chinense*	15	19	8	12	10	13	13	10	19	11	+
H12	31	*L. chinense*	15	18	8	12	10	13	15	10	19	11	+
H13	1	*L. chinense*	16	17	8	12	10	13	13	10	19	11	+
H14	12	*L. chinense*	16	18	8	12	10	14	13	10	19	11	+
H15	7	*L. chinense*	16	19	8	13	10	13	15	10	19	11	+
H16	27	*L. chinense*	17	18	8	12	10	13	13	10	19	11	+
H17	20	*L. chinense*	18	18	8	12	10	13	13	10	19	11	+
H18	16	*L. chinense*	18	17	8	11	10	13	14	10	19	11	+
H19	33	*L. chinense*	16	18	8	12	10	13	13	10	20	11	+
H20	16	*L. chinense*	16	17	8	11	10	13	14	10	19	12	+
H21	1	*L. chinense*	18	17	8	11	10	13	14	10	19	12	+
H22	26	*L. chinense*	18	15	8	11	10	14	11	10	26	12	+
H23	1	*L. chinense*	14	15	8	12	10	13	12	10	29	13	+
H24	15	*L. chinense*	15	15	8	12	10	13	12	10	29	13	+
H25	33	*L. chinense*	15	15	8	12	10	13	10	10	30	13	+
H26	5	*L. chinense*	18	15	8	12	11	13	12	10	27	12	+
H27	10	*L. chinense*	11	18	8	11	11	13	13	10	20	13	+
H28	19	*L. chinense*	18	19	8	12	11	13	12	10	23	14	+
H29	1	*L. chinense*	18	19	8	13	11	13	12	10	23	14	+
H30	1	*L. chinense*	22	14	8	11	11	14	9	10	24	14	+
H31	2	*L. chinense*	24	14	8	11	11	14	9	10	24	14	+
H32	43	*L. chinense*	23	20	8	12	11	12	12	10	25	14	+
H33	3	*L. chinense*	22	14	8	12	11	13	9	10	29	14	+

Haplotype	No. indiv.	Species	Repeat numbers of each locus										
			Lcp5	Lcp15	Lcp19	Lcp21	Lcp24	Lcp26	Lcp33	Lcp39	Lcp48	Lcp49	Lcp5-2
H34	24	*L. chinense*	19	18	8	10	12	14	16	10	20	12	+
H35	5	*L. chinense*	12	18	8	11	12	13	13	10	19	13	+
H36	5	*L. chinense*	12	18	8	11	12	13	13	10	20	13	+
H37	8	*L. chinense*	20	13	8	10	10	12	15	11	21	12	+
H38	9	*L. chinense*	20	13	8	10	10	12	16	11	21	12	+
H39	13	*L. chinense*	20	13	8	10	10	12	15	11	21	13	+
H40	4	*L. chinense*	19	13	8	10	10	12	15	11	22	13	+
H41	8	*L. chinense*	15	13	8	10	10	12	14	11	21	14	+
H42	4	*L. chinense*	16	16	9	11	10	12	9	9	18	12	–
H43	6	*L. chinense*	17	16	9	11	10	12	9	9	18	12	–
H44	25	*L. chinense*	18	16	9	11	10	12	9	9	18	12	–
H45	1	*L. chinense*	18	16	9	11	10	12	9	9	19	12	–
H46	13	*L. chinense*	16	16	9	11	10	12	9	9	18	13	–
H47	25	*L. chinense*	17	22	9	11	10	11	9	9	18	13	–
H48	21	*L. chinense*	17	16	9	11	10	12	10	9	18	13	–
H49	6	*L. chinense*	16	21	9	11	10	11	9	9	18	14	–
H50	1	*L. tulipifera*	12	11	9	14	10	10	9	12	33	14	–
H51	13	*L. tulipifera*	13	11	9	14	10	10	9	12	33	14	–
H52	1	*L. tulipifera*	13	11	9	14	10	10	10	12	33	14	–
H53	1	*L. tulipifera*	14	11	9	14	10	10	9	12	33	14	–
H54	1	*L. tulipifera*	11	11	9	14	11	10	10	12	32	14	–
H55	1	*L. tulipifera*	11	11	9	14	11	10	9	12	33	14	–
H56	3	*L. tulipifera*	11	11	9	14	11	10	10	12	33	14	–
H57	1	*L. tulipifera*	11	11	9	14	11	10	11	12	33	14	–
H58	1	*L. tulipifera*	11	11	9	14	11	10	9	12	34	14	–
H59	1	*L. tulipifera*	11	11	9	14	11	10	10	12	34	14	–
H60	1	*L. tulipifera*	12	11	9	14	11	10	11	12	34	14	–
H61	1	*L. tulipifera*	13	11	9	14	11	10	10	12	34	14	–
H62	1	*L. tulipifera*	11	11	9	14	11	10	10	12	33	15	–

Supplementary Table S2 Estimates of genetic variation parameters based on ten cpSSR and eight nSSR loci in the 29 *Liriodendron chinense* populations, and genetic diversity comparison between central and peripheral populations.

Population	Group	N_T	cpSSR					nSSR						
			N_A	N_E	H_R	H	A_P	N_A	N_e	A_R	H_O	H_E	A_p	F_{IS}
JP	S	600	2	1.125	1.588	0.118	2	3.125	1.714	2.327	0.176	0.339	2	0.62
MLP	S	50	1	1.000	1.000	0.000	1	2.25	1.497	1.840	0.219	0.268	0	0.232
WM	S	400	1	1.000	1.000	0.000	1	4	2.661	3.157	0.532	0.539	0	0.015
LB	S	100	1	1.000	1.000	0.000	1	3.25	2.045	2.588	0.466	0.451	0	–0.048
DY	S	300	2	1.180	1.697	0.159	1	3.625	2.385	3.049	0.525	0.510	0	–0.047
JH	C	2000	1	1.000	1.000	0.000	1	4.75	3.005	3.492	0.506	0.606	0	0.24
XF	C	50	2	1.800	2.000	0.389	0	2.875	1.985	2.649	0.384	0.426	0	0.109
PA	S	1000	1	1.000	1.000	0.000	1	2.5	1.523	1.841	0.301	0.258	1	–0.109
YJ	C	100	3	2.667	2.969	0.658	3	5	3.106	3.692	0.448	0.542	0	0.181
XY	C	600	1	1.000	1.000	0.000	1	3.125	2.166	2.585	0.391	0.442	2	0.108
TZ	C	500	2	1.142	1.648	0.133	1	4.75	3.191	3.907	0.520	0.639	0	0.173
NC	C	400	1	1.000	1.000	0.000	0	5.25	2.817	3.683	0.365	0.567	0	0.336
SW	N	400	1	1.000	1.000	0.000	1	2.75	1.816	2.124	0.483	0.409	0	–0.150
SNJ	N	300	2	1.385	1.931	0.294	2	3.375	2.116	2.693	0.563	0.509	0	–0.085
JS	N	100	2	1.600	1.985	0.395	2	8	4.745	5.185	0.506	0.699	1	0.232
YY	C	600	4	2.613	3.194	0.641	2	7	4.129	4.692	0.624	0.695	0	0.099
K	C	300	2	1.324	1.868	0.252	0	7.25	3.895	4.501	0.631	0.648	3	0.018
ST	C	500	2	1.835	1.998	0.479	1	4.25	3.096	3.530	0.574	0.623	0	0.091
ZY	S	500	3	1.770	2.382	0.450	3	5.87	3.790	4.230	0.620	0.720	0	0.124
HGS	C	100	3	2.571	3.000	0.600	2	6	3.006	4.750	0.563	0.574	1	0.066
JLS	C	100	3	2.740	2.949	0.667	2	7.125	4.756	5.001	0.672	0.714	3	0.070
BSZ	C	300	3	2.740	2.949	0.667	2	7	3.776	4.617	0.615	0.626	0	0.017
ZR	C	50	1	1.000	1.000	0.000	1	1.625	1.576	1.625	0.307	0.300	1	–0.030
SRQ	N	200	1	1.000	1.000	0.000	1	5.625	3.584	4.083	0.551	0.633	0	0.236
DWD	N	200	2	1.438	1.937	0.315	2	7.25	4.789	4.872	0.660	0.708	0	0.052
WFS	N	300	3	2.305	2.915	0.593	2	4.625	3.216	3.645	0.506	0.532	0	0.079
JGS	C	500	3	1.163	1.797	0.145	2	4.25	2.492	3.202	0.623	0.549	0	–0.104
LS	C	100	2	1.454	1.944	0.323	2	5.5	3.398	3.957	0.569	0.612	0	0.076
SY	C	100	1	1.000	1.000	0.000	1	3	2.039	2.411	0.387	0.460	0	0.183
Mean	M	342.308	1.692	1.293	1.572	0.179	1.538	4.327	2.761	3.203	0.470	0.506	0.308	0.089
	C	393.750	2.125	1.690	1.957	0.310	1.313	4.922	3.027	3.643	0.511	0.564	0.625	0.102
p		0.728	0.195	0.090	0.180	0.171	0.576	0.374	0.483	0.265	0.409	0.255	0.713	0.822

NOTE: S: south marginal population; N: north marginal population; C: central population; N_A: number of different haplotypes; N_E: number of effective haplotypes; H_R: haplotype richness; H: haplotype diversity; A_P: private haplotypes; N_a: number of different alleles; N_e: number of effective Alleles; H_O: observed heterozygosity; H_E: expected heterozygosity; A_p: number of private alleles; F_{IS}: Fixation index.

Supplementary Table S3 Analysis of molecular variance (AMOVA) for chloroplast and nuclear microsatellite data of 29 *Liriodendron chinense* populations.

Source of variation	d.f.	Sum of squares	Vairiance components	% Total variation	*p*-value	F_{ST}
Chloroplast microsatellite						
Among groups	1	366.651	0.972	25.67	< 0.001	
Among Populations within groups	27	1581.709	2.453	64.82	< 0.001	
Within populations	664	238.796	0.360	9.50	< 0.001	
Total	692	2187.156	3.785			0.905^*
Nuclear microsatellite						
Among groups	1	88.784	0.063	2.02	< 0.001	
Among Populations within groups	27	1218.488	0.905	28.81	< 0.001	
Within populations	1357	2948.942	2.173	69.17	< 0.001	
Total	1385	4256.214	3.142			0.308^*

d.f., degree of freedom; * $p < 0.001$.

Supplementary Table S4 Total variance explained in principal component analysis using nSSR.

	Initial Eigenvalues			Extraction Sums of Squared Loadings		
Component	Total	% of Variance	Cumulative %	Total	% of Variance	Cumulative %
1	3.789	94.728	94.728	3.789	94.728	94.728
2	.172	4.292	99.020			
3	.029	0.717	99. 737			
4	.011	.263	100.000			

Supplementary Table S5. Total variance explained in principal component analysis using cpSSR.

	Initial Eigenvalues			Extraction Sums of Squared Loadings		
Component	Total	% of Variance	Cumulative %	Total	% of Variance	Cumulative %
1	4.506	90.116	90.116	4.506	90.116	90.116
2	.362	7.241	97.357			
3	.071	1.424	98.781			
4	.045	.908	99.689			
5	.016	.311	100.000			

Supplementary Method S1

The raggedness index (r) [1] and the sum of square deviation (SSD) between observed and expected mismatch distributions [2] were calculated to test the demographic expansion hypothesis using Arlequin version 3.1 [3]. In the neutral test, Fu's *F*s [4] value were calculated and permuted 1 000 times to test the sudden expansion hypothesis.

References:

1. Harpending, H.C. Signature of ancient population growth in a low-resolution mitochondrial DNA mismatch distribution. *Human Biology*, **66**, 591–600 (1994).
2. Schneider, S., Excoffier, L. Estimation of past demographic parameters from the distribution of pairwise differences when the mutation rates vary among sites: application to human mitochondrial DNA. *Genetics*, **152**, 1079–1089 (1999).
3. Excoffier, L., Laval, G., Schneider, S. Arlequin (version 3.0): an integrated software package for population genetics data analysis. *Evol. Bioinform. Online* **1**, 47-50 (2005).
4. Fu, Y.X. Statistical tests of neutrality of mutations against population growth, hitchhiking and background selection. *Genetics*, **147**, 915–925 (1997).
